# Relaxin Attenuates Contrast-Induced Human Proximal Tubular Epithelial Cell Apoptosis by Activation of the PI3K/Akt Signaling Pathway In Vitro

**DOI:** 10.1155/2017/2869405

**Published:** 2017-04-30

**Authors:** Xiang-Cheng Xie, Yizhi Cao, Xiu Yang, Qun-Hong Xu, Wei Wei, Ming Wang

**Affiliations:** ^1^Department of Nephrology, Hangzhou First People's Hospital, Nanjing Medical University, Hangzhou, Zhejiang, China; ^2^Department of Pathophysiology, Nanjing Medical University, Nanjing, Jiangsu, China

## Abstract

*Background*. Contrast-induced acute kidney injury (CI-AKI) is one of the main causes of iatrogenic acute kidney injury (AKI); however, therapeutic strategies for AKI remain limited. This study aims to explore the effect of relaxin (RLX) on contrast-induced HK-2 apoptosis and its underlying mechanisms.* Methods*. Renal tubular epithelial cells (HK-2) were incubated either with or without ioversol, human H2 relaxin, and LY294002 (the inhibitor of the PI3K/Akt signal pathway). Cell viability was evaluated with a CCK-8 assay. Apoptotic morphologic alterations were observed using the Hoechst 33342 staining method. Apoptosis was detected with Annexin V staining. Western blot analysis was employed to measure the expression of pAkt (S473), Akt, cleaved caspase-3, Bcl-2, Bax, and actin proteins.* Results*. Ioversol reduced the viability of HK-2 cells. Western blotting results revealed decreased expression of phosphorylated Akt in cells treated with ioversol. The activities of caspase-3 and Bax protein increased, while the expression of Bcl-2 protein decreased. As a result, the Bax/Bcl-2 ratio increased after treatment with ioversol. These effects were reversed when HK-2 cells were cotreated with RLX. However, with preadministration of PI3K/Akt pathway inhibitor LY294002, the effect of RLX was blocked.* Conclusion*. Our study demonstrates that relaxin attenuates ioversol induced cell apoptosis via activation of the PI3K/Akt signaling pathway, suggesting that RLX might play a protective role in the treatment of CI-AKI.

## 1. Introduction

Iodinated contrast agents are widely used in radiographic diagnostic and interventional procedures, and their toxic effects on renal function have also been recognized. The incidence of contrast-induced acute kidney injury (CI-AKI) ranges from 10% to 30%, depending on the criteria employed for CI-AKI [1, 2]. CI-AKI is now the third most frequent cause of hospital-acquired kidney dysfunction [3]. CI-AKI has been shown to be associated with an increased risk of mortality, increased costs of medical care, and prolonged hospitalization [4, 5].

The pathophysiology of CI-AKI, which depends on the concentration of contrast within the kidney, involves vasoconstriction mediated by endothelin, inhibition of nitric oxide, reduced renal blood flow, the induction of oxygen free radicals through oxidative stress, and direct toxic effects to the tubular cells. These factors eventually lead to tubular epithelial injury [6]. Contrast agents also cause direct tubulotoxicity that can lead to mitochondrial damage, the generation of reactive oxygen species, and apoptosis [7]. There is evidence suggesting that cell apoptosis plays a crucial role in the development of AKI [8]. Previous studies have shown that iodinated contrast agents can directly induce apoptosis in renal tubular cells [9–11].

To date, there is no specific treatment for CI-AKI; thus, the most effective strategy is prevention [12]. However, although strategies for preventing CI-AKI have been extensively studied, pharmacological renal protection strategies still need to be explored [13].

Human H2 relaxin (RLX), which belongs to the relaxin/insulin peptide hormone family, has been shown to possess antiapoptotic effects. Evidence shows that relaxin plays a beneficial role in ischemia-reperfusion-induced renal injury through the inhibition of apoptosis [14]. In another study, RLX was found to reduce H_2_O_2_-induced cardiomyocyte apoptotic death by activation of the Akt signal pathway [15].

Based on these findings, we hypothesized that RLX may also reduce contrast agent-induced tubular epithelial cell apoptosis. Therefore, this study aims to investigate whether RLX can mitigate contrast-induced tubular epithelial cell apoptosis by activation of the PI3K/Akt signaling pathway.

## 2. Materials and Methods

### 2.1. Cell Culture

Human proximal tubular epithelial cells (HK-2, Cell Bank of the Chinese Academy of Sciences) were cultured in 1 : 1 DMEM/F12 (Gibco, Invitrogen, USA) medium supplemented with 10% fetal bovine serum (Sijiqing Hangzhou, China) in 5% CO2 humidified air at 37°C. The medium was shifted every 2~3 days until the cells were approximately 80% confluent. Cells were arrested by 24-hour incubation in a serum free medium and subsequently exposed to various agents.

### 2.2. Cell Viability Assay

HK-2 cells were seeded in 96-well plates (Corning Incorporated) at a concentration of 4 × 10^3^ cells per well. Cells were then exposed to ioversol (25 to 150 mg iodine/mL) for 30 minutes. After exposure to ioversol, the cells were washed twice using Krebs-Ringer solution and then incubated in DMEM/F12 medium with 10% fetal bovine serum for 24 hours, 48 hours, and 72 hours at 37°C in 5% CO_2_ air. Cell viability was detected using a cell counting Kit-8 kit (CCK-8, Dojindo, Japan) in accordance with the manufacturer's protocol.

After the above results were analyzed, the intervention concentration of ioversol (100 mg iodine/mL, Hengrui Medicine Co., Ltd., China) was selected. To test the effects of recombinant human H2 relaxin (Protech, USA) and LY294002 on contrast-induced cell viability, HK-2 cells were exposed to RLX (10 ng/mL) and LY294002 (50 *μ*M, Selleck Chemicals) for 1.5 hours and then treated with ioversol. The absorbance was measured at 450 nm using a microplate reader (Tecan, Durham, NC).

### 2.3. Annexin V and Propidium Iodide Staining

Cells were seeded onto 6-well plates and treated with different agents. Cells were collected with trypsin. The harvested cells were then centrifuged at 2000 RPM for 5 minutes, washed twice with PBS solution, and suspended at a concentration of 1 × 10^6^ cells/mL with 400 *μ*L 1x binding buffer (M3036), and then, 5 *μ*L Annexin V-FITC (M3031, Mbchem) was added and incubated in the dark for 15 min. 10 *μ*L propidium iodide (PI, M3032, Mbchem) was added. The features of apoptotic cells were detected by flow cytometry (FACS Arial II, BD, Biosciences, USA).

### 2.4. Apoptotic Morphological Observation

HK-2 cells were seeded in 6-well plates. The cells were pretreated with RLX (10 ng/mL) and LY294002 (50 *μ*M) for 1.5 hours before ioversol was added and then cultured for 24 hours. Next, the cells were fixed with Carnoy's solution, washed twice with 0.01 mol/L PBS for 5 minutes, and then incubated with Hoechst 33342 at a concentration of 0.5 *μ*g/mL (Beyotime, China) for 15 minutes. The incubation occurred at room temperature, and the plates were kept in a dark place. Finally, the cells were washed three times with 0.01 mol/L PBS, placed on microscopic slides with cover slips, and examined with an Olympus fluorescence microscope for morphological changes.

### 2.5. Western Blot Analysis

Cells cultured in 6-well plates were harvested with trypsin and centrifuged at 1,500 rpm for 5 minutes at 25°C. Afterwards, the cells were suspended and centrifuged again at 1,500 rpm at 25°C for 5 minutes and then washed twice with PBS. Next, the cells were lysed with RIPA Lysis Buffer (Beyotime, China) following a centrifugation at 12,000 rpm for 30 minutes at 4°C. The samples were then boiled for 5 minutes in 4x SDS loading buffer. Subsequently, equal amounts of total cellular proteins were electrophoresed in 8% w/v sodium dodecyl sulfate-polyacrylamide gel (SDS-PAGE) at 80 V for 30 minutes and 120 V for 70 minutes. The proteins were then transferred to polyvinylidene difluoride membranes (PVDF) at a constant 220 mA for 90 min. The membranes were blocked with a blocking buffer (1x PBS, 0.1% Tween-20, and 5% w/v nonfat milk) for 1 hour at room temperature. After being rinsed in TBS-T three times, the membranes were incubated overnight at 4°C with the primary antibodies anti-total Akt, anti-phospho-Akt (Affinity Biosciences, USA), anti-caspase-3, anti-Bax, anti-Bcl-2, and anti-*β*-catenin (Proteintech, America). After three-wash cycles with TBS-T, the blots were incubated with horseradish peroxidase-labeled-conjugated secondary antibodies (Proteintech, USA) for 2 hours before the membranes were washed in TBS-T again and developed using an enhanced chemiluminescent reporter system detection kit. Protein bands were quantified with TINA image software (Raytest, Straubenhardt, Germany).

### 2.6. Statistical Analysis

All values are expressed as the mean ± standard deviation (SD). One way analysis of variance (ANOVA) with a post hoc Student-Newman-Keuls multiple comparisons test was performed to evaluate significant differences using SPSS Software (V21.0, SPSS, Inc., Chicago, IL, USA). Values of *P* < 0.05 were considered statistically significant.

## 3. Results

### 3.1. RLX Alleviates Contrast-Induced Injury to HK-2 Cells

**Figure 1:**
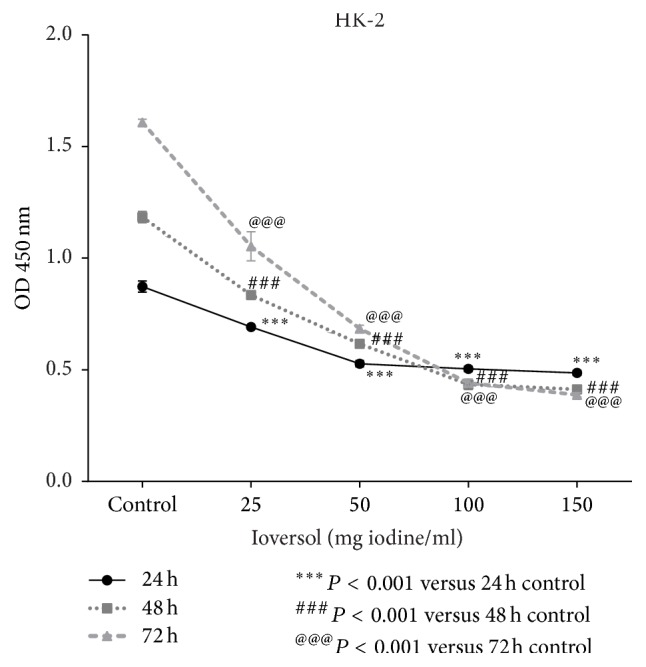
Ioversol cell inhibits cell proliferation in a time- and dose-dependent manner. HK-2 cells were exposed to 25, 50, 100, and 150 mg/mL ioversol for 30 minutes and then incubated without ioversol for 24 h, 48 h, and 72 h. Survival rates were examined using CCK-8 assay kit. The values are representative of three independent experiments.

**Figure 2:**
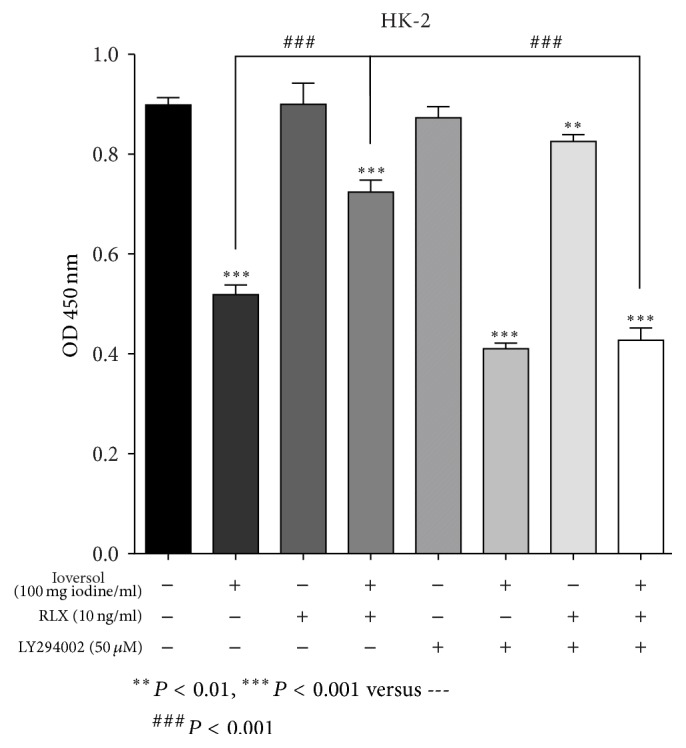
RLX alleviates the ioversol induced cytotoxicity in HK-2 cells. We further examined the effect of H2-RLX on ioversol (100 mg/mL) treated HK-2 cells at 24 h using a CCK-8 assay. The concentrations employed were based on the previous results. HK-2 cells were pretreated with RLX (10 ng/mL) and LY294002 (50 *μ*M) for 1.5 hours before ioversol was added. And then cells were exposed to ioversol (100 mg iodine/mL) for 30 minutes, followed by further incubation for 24 hours in the absence of ioversol. Data are reported as the mean ± SD; *n* = 3.

The reduction in viability of HK-2 cells exposed to ioversol is both dose-dependent and time-dependent, as reflected in the results for the CCK-8 kit assay (Figure 1). These results suggest that ioversol may cause cytotoxic effects in tubular epithelial cells, while RLX might mitigate this cytotoxic effect. However, the protective role of RLX was suppressed in the presence of the PI3K/Akt pathway inhibitor LY294002 (Figure 2).

### 3.2. Impacts of Ioversol and RLX on Apoptosis in HK-2 Cells

**Figure 3:**
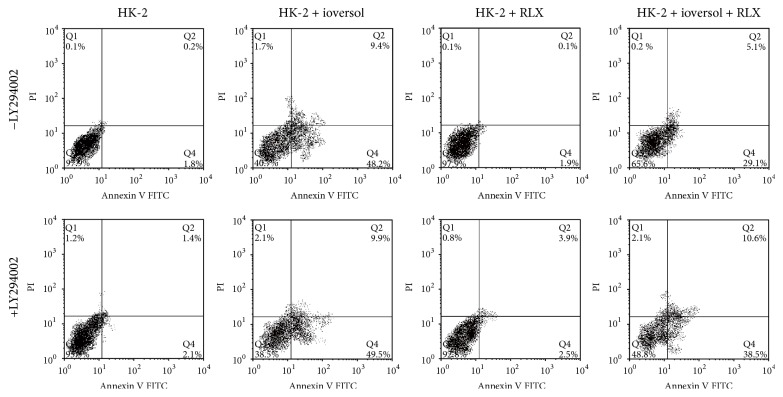
RLX reduces ioversol induced apoptosis. HK-2 cells were pretreated with RLX (10 ng/mL) and LY294002 (50 *μ*M) for 1.5 hours and then coincubated with ioversol (100 mg iodine/mL) for 30 minutes, followed by further incubation for 24 hours in the absence of ioversol. Cell apoptosis was analyzed by flow cytometry using Annexin V-FITC and PI staining. Data showed a marked increase of apoptotic cells in the ioversol treated cells; however, apoptotic cells were reduced after treatment with RLX; this effect was inhibited by the PI3K/Akt pathway inhibitor LY 294002.

Based on the previous results, 100 mg iodine/mL ioversol and 10 ng/mL RLX were used to treat HK-2 cells. HK-2 cells treated with ioversol for 24 hours showed a marked increase in apoptotic cells compared to control cells; when cotreated with RLX, the number of apoptotic cells was significantly reduced. However, when the PI3K/Akt inhibitor LY 294002 was present at a concentration of 50 *μ*M, the protective effect of RLX was significantly reduced (Figure 3).

**Figure 4:**
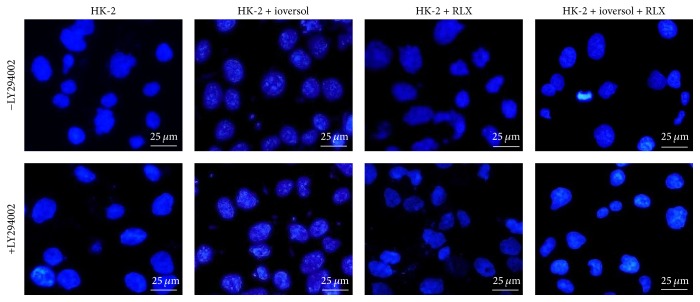
Morphological alterations of ioversol induced cell apoptosis. RLX (10 ng/mL) was added 1.5 hours before ioversol treatment and was in the presence throughout the experiment. HK-2 cells were subjected to ioversol (100 mg/mL) for 30 minutes, and then ioversol was removed, followed by further incubation for 24 hours. Apoptosis was determined using Hoechst 33258 staining (magnification, ×400). Compared with the control, cells treated with ioversol (100 mg/mL) exhibited shrunken nuclear and chromatin condensation, while less apoptotic morphology changes were observed in RLX treated cells.

The morphological apoptotic changes in HK-2 cells induced by ioversol were observed using the Hoechst 33258 staining method. These apoptotic morphological alterations consisted of bright blue apoptotic nuclei, highly condensed chromatin, and the appearance of apoptotic bodies. After treatment with RLX, the toxic effects of contrast diminished (Figure 4); however, the antiapoptotic effect of RLX was markedly suppressed when the cells were coincubated with the PI3K/Akt inhibitor, LY294002.

### 3.3. Effects of RLX on Expression of Akt, Caspase-3, Bax, and Bcl-2

**Figure 5:**
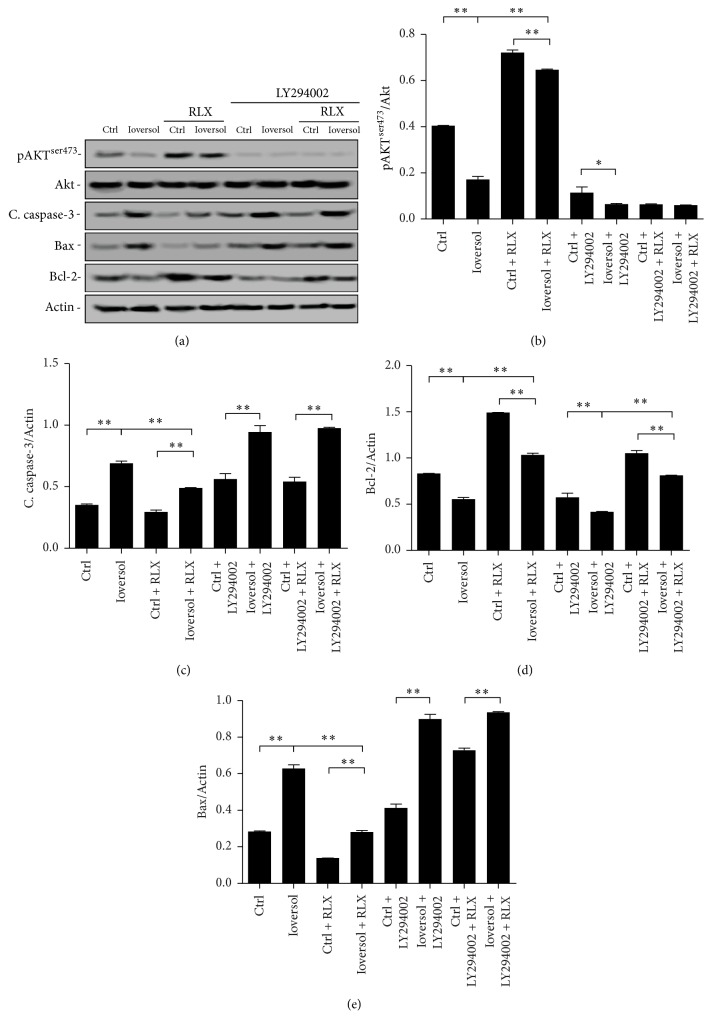
Effects of RLX on the expression of pAkt and apoptosis proteins. RLX (10 ng/mL) or LY294002 (50 *μ*M) was added 1.5 hours before ioversol treatment. Cells were then exposed to ioversol (100 mg/mL) for 30 minutes and were further incubated for 24 hours in the absence of ioversol. The expression of pAkt was significantly lower in ioversol treated cells compared with the control, which could be reversed by treatment of RLX. Furthermore, this effect was attenuated by Akt inhibitor LY294002. Expression of cleaved caspase3 was markedly increased following the exposure to ioversol, whereas cleaved caspase-3 decreased when cotreated with RLX. This antiapoptotic effect was abrogated by the PI3K/Akt inhibitor LY294002. Bcl-2 expression was significantly reduced in the ioversol treated HK-2 cells; while coincubated with RLX, the expression of Bcl-2 was restored. The expression of Bax showed a reverse association with that of Bcl-2. The ratio of Bax/Bcl-2 decreased in RLX treated cells compared to control or ioversol groups. Experiments were repeated three times. ^*∗*^*P* < 0.05; ^*∗∗*^*P* < 0.01.

Western blotting was performed to analyze the possible mechanisms by which RLX exerts an antiapoptotic effect via the PI3K-Akt signaling pathway. The expression of pAkt (S473) in cells treated with ioversol was markedly decreased. In contrast, RLX significantly upregulated the expression of pAkt in ioversol treated cells (Figure 5(a)). With coadministration with ioversol, RLX, and the PI3K/Akt inhibitor LY294002 (50 *μ*M), the expression of pAkt (S473) was significantly decreased, suggesting that the inhibition of Akt can prevent RLX from exerting its antiapoptotic effect through the PI3K/Akt signaling pathway (Figure 5(b)).

As shown in Figure 5(c), ioversol induced a marked increase in caspase-3 activity, while RLX to some extent reversed the increased expression of caspase-3 induction by ioversol. However, the antiapoptotic effect of RLX can be inhibited by LY294002. Expression of Bcl-2 in cells treated with ioversol was significantly decreased compared to the control group, and the expression of Bcl-2 was restored after treatment with ioversol and RLX. However, the antiapoptotic effect of RLX was inhibited by the PI3K/Akt inhibitor LY294002 (Figure 5(d)). In contrast, Bax expression showed an inverse association with that of Bcl-2 (Figure 5(e)). These results suggest that RLX may exert an antiapoptotic effect through the PI3K/Akt signaling pathway.

## 4. Discussion

In the present study, we investigated whether RLX can reduce ioversol induced apoptosis by upregulating the PI3K/Akt pathway. Our results demonstrate that ioversol induces a marked increase in apoptosis in HK-2 cells by upregulating the cleaving of caspase-3 and increasing the Bax/Bcl-2 ratio. In contrast, RLX exhibits a protective action against ioversol induced apoptosis by promoting the PI3K/Akt signaling pathway.

In the article published by Hardiek et al. [16], iopamidol was used, while in our study, ioversol was used. Both iopamidol and ioversol are nonionic low-osmolar radiocontrast media and are similar. In Hardiek et al.'s article, the maximum concentration and incubation time were 100 mg iodine/mL and 60 h, respectively, while in our study, the maximum concentration and incubation time were 150 mg iodine/mL and 72 h, which were employed for screening of the intervention conditions; after the screening, 100 mg iodine/mL and 24 h-time point were used. Therefore, the concentration of ioversol and its time of incubation with the cells in our study were comparable to the previous report.

The pathogenesis of CI-AKI involves increased adenosine, endothelin, and renal vasoconstriction, oxygen free radical formation secondary to oxidative stress, reduced nitric oxide, and direct tubular toxicity. These mechanisms result in altered mitochondrial function and apoptosis. There is compelling evidence demonstrating that apoptosis plays a pivotal role in the progression of CI-AKI [7, 17, 18].

In a previously published study investigating the effects of the low-osmolar radiocontrast agent iomeprol, HK-2 cells were incubated with a concentration of 100 mg iodine/mL iomeprol for an initial 3-hour period followed by removal of the radiocontrast agent and allowing the cells to incubate for a further 21 h and the cells showed a complete recovery of cell viability and a recovery in pAkt levels [19]. This result indicates that the iomeprol induced cell damage is not permanent but reversible. Because both iomeprol and ioversol are low-osmolar radiocontrast media, we thought that ioversol might have the similar effects.

In this study, the pretreatment with RLX did not lead to full recovery in cell viability, which might be due to the involvement of other pathways/molecules not investigated in the present study, like Erk-1/2, JNKs, p38, NF-*ƙ*B, and so forth [20]. Moreover, in the article published by Michele Andreucci, the authors found that the transfection of HK2 cells with a constitutively active Akt plasmid led to partial recovery of cell viability [21], which demonstrated the protective effects of Akt during the cell injury by radiocontrast. This in turn supports our findings about the protective role of Akt in contrast-induced cell apoptosis.

The intrinsic mitochondrial apoptotic pathway functions mainly through Bcl-2 family proteins by altering the permeability of the outer mitochondrial membrane. These proteins include proapoptotic (Bax, Bak, Bid, Bad, and Bok) and antiapoptotic members (Bcl-2, Bcl-x, Bcl-xL, Mcl-1, and Bcl-w). An increase in Bcl-2 enhances the antiapoptotic function of cells, whereas an overexpression of Bax accelerates cell apoptosis. Additionally, Bcl-2 can also form a heterodimer with Bax that regulates the release of cytochrome C from mitochondria. Cytochrome c released from mitochondria binds to cytosolic Apaf-1 and procaspase-9, forming apoptosomes, which subsequently activate caspase-9 and caspase-3. These proteases are the executors that cleave key cellular proteins, resulting in cell apoptosis. As been well documented, the ratio of Bax/Bcl-2 in the plasma membrane reflects the level of cell apoptosis [22, 23]. Our findings show that Bcl-2 expression increased in RLX treated cells, whereas that of Bax decreased so that the Bax/Bcl-2 ratio decreased, resulting in improved antiapoptotic effects. Our results showed a marked reduction in the cleaved form of caspase-3 in RLX treated cells, indicating that RLX may alleviate contrast-induced tubular epithelial cell apoptosis by suppressing the activity of caspase-3.

RLX, a two-chain peptide hormone, belongs to the relaxin peptide family, which is structurally similar to the insulin family. RLX exerts its bioeffect by activating the relaxin family peptide receptors (RXFP1, RXFP2, RXFP3, and RXFP4) [24]. RLX has been found to be involved in the pathophysiology of hypertension, heart failure, fibrosis, angiogenesis, and bone remodeling [25]. RLX was previously reported to alleviate cisplatin-caused renal tubular cell apoptosis by increasing the Bcl-2/Bax ratio [14]. RLX was also found to play a protective role in ischemic reperfusion-induced renal injury in rats via both antiapoptotic and anti-inflammatory mechanisms [26]. Recombinant RLX was shown to reduce renal interstitial fibrosis in several experimental models of renal disease [27]. In addition, serelaxin, a recombinant form of RLX-2, was found to improve heart failure and renal function impairment in patients with AKI [28].

Previous animal experiments have revealed that the phosphorylation of Akt increases while the expression of Bad decreases in mouse kidneys subjected to ischemia/reperfusion [29]. Our results show that phosphorylated Akt is reduced by treatment with ioversol in HK-2 cells but is increased by treatment with RLX. Furthermore, this effect can be blocked by the presence of the PI3K/Akt inhibitor LY294002 indicating that the antiapoptotic effect of RLX is mediated at least in part by the upregulation of the PI3K/Akt signaling pathway. Mounting evidence demonstrates that the PI3K/Akt signaling pathway participates in cell survival, proliferation, growth, and angiogenesis [30, 31]. Akt phosphorylates Bad and subsequently the phosphorylated forms of Bad bind with 14-3-3 protein, which in turn prevents Bad from associating with BCL-XL or BCL-2 [32]. Therefore, the phosphorylation of Bad suppresses apoptosis and promotes cell survival [33, 34].

In conclusion, our study shows that H2 relaxin can inhibit ioversol induced apoptosis, possibly via activation of the PI3K/Akt signaling pathway. This suggests that relaxin may have a protective role in the treatment of CI-AKI. Further in vivo experiments are required to confirm its renal protective role.
